# Prescribers’ satisfaction with delivering medications for opioid use disorder

**DOI:** 10.1186/s13011-021-00413-7

**Published:** 2021-10-18

**Authors:** Hannah K. Knudsen, Randy Brown, Nora Jacobson, Julie Horst, Jee-Seon Kim, Hanna Kim, Lynn M. Madden, Eric Haram, Todd Molfenter

**Affiliations:** 1 Department of Behavioral Science and Center on Drug and Alcohol Research, University of Kentucky, 845 Angliana Ave., Room 204, KY 40508 Lexington, USA; 2 Department of Family Medicine and Community Health, University of Wisconsin-Madison, 1100 Delaplaine Ct, WI 53715-1896 Madison, USA; 3 Institute for Clinical and Translational Research, University of Wisconsin-Madison, 4116 Signe Skott Cooper Hall, 701 Highland Ave, WI 53705 Madison, USA; 4 Department of Industrial and Systems Engineering, University of Wisconsin-Madison, 1513 University Ave, WI 53706 Madison, USA; 5 Department of Educational Psychology, University of Wisconsin-Madison, 1025 West Johnson St, WI 53706-1706 Madison, USA; 6 Department of Internal Medicine, Yale University, APT Foundation, 1 Long Wharf Drive, Suite 321, CT 06511 New Haven, USA; 7 Haram Consulting, 413 River Road, ME 04008 Bowdoinham, USA

**Keywords:** prescriber satisfaction, buprenorphine, extended-release naltrexone, opioid use disorder

## Abstract

**Background:**

Expanding access to medications for opioid use disorder (MOUD), such as buprenorphine and extended release (XR) naltrexone, is critical to addressing the US opioid epidemic, but little is known about prescriber satisfaction with delivering these two types of MOUD. The current study describes the satisfaction of prescribers delivering buprenorphine and XR-naltrexone while examining whether satisfaction is associated with current patient census and organizational environment.

**Methods:**

As part of a cluster randomized clinical trial (RCT) focused on expanding access to medication for opioid use disorder, 41 MOUD prescribers in Florida, Ohio, and Wisconsin completed a web-based survey. The survey included measures of prescriber satisfaction with delivering buprenorphine treatment and XR-naltrexone. In addition, the survey measured several prescriber characteristics and their perceptions of the organizational environment.

**Results:**

Prescribers were generally satisfied with their work in delivering these two types of MOUD. Prescribers reporting a greater number of patients (*r* = .46, *p* = .006), those who would recommend the center to others (*r* = .56, *p* < .001), and those reporting positive relationships with staff (*r* = .56, p < .001) reported significantly greater overall satisfaction with delivering buprenorphine treatment. Prescribers who more strongly endorsed feeling overburdened reported lower overall buprenorphine satisfaction (*r* = -.37, *p* = .02). None of the prescriber characteristics or perceptions of the organizational environment were significantly associated with overall satisfaction with delivering XR-naltrexone treatment.

**Conclusions:**

The generally high levels of satisfaction with both types of MOUD is notable given that prescriber dissatisfaction can lead to turnover and impact intentions to leave the profession. Future research should continue to explore the prescriber characteristics and organizational factors associated with satisfaction in providing different types of MOUD.

**Registration:**

ClinicalTrials.gov. NCT02926482. Date of registration: September 9, 2016. https://clinicaltrials.gov/ct2/show/NCT02926482.

## Background

The US opioid epidemic continues to be a major public health problem, resulting in nearly 450,000 deaths from 1999 to 2018 [1], with concerns growing amid early evidence that the COVID-19 pandemic is worsening this crisis [2–4]. Limited access to evidence-based treatment, particularly medications for opioid use disorder (MOUD) [5, 6], continues to be a significant challenge despite efforts to expand treatment access [7–9]. If access to MOUD is to expand in the US, it is critically important to continue to expand the number of medical professionals who are willing to prescribe buprenorphine and extended-release naltrexone (XR-naltrexone). Although the number of medical professionals who have the capacity to prescribe buprenorphine continues to grow [10–13], recent data indicate that only half of waivered prescribers have any patients receiving buprenorphine and that median monthly census is considerably lower than the cap on patients, even among those holding the 275-patient waiver [14]. Utilization of XR-naltrexone is even lower than buprenorphine [15], particularly in primary care [16].

An understudied issue is prescriber satisfaction with delivering these two types of MOUD. A systematic review published by Becker and Fiellin in 2005 [17] noted a gap in the literature regarding prescriber satisfaction with delivering buprenorphine treatment. Since that review, there have been two publications regarding prescriber satisfaction, one a qualitative study of rural prescribers [18] and the other a survey of a small sample of physicians in three states [19]. Andrilla and colleagues found that rural prescribers described their buprenorphine practice as rewarding and meaningful. However, a survey of waivered physicians indicated that prescribers rated their buprenorphine work as significantly less personally rewarding when compared to their general medical practice [19]. To date, there have been no studies of prescriber satisfaction with delivering XR-naltrexone as a treatment for OUD. These gaps in the literature are notable because there is a substantial literature regarding global physician satisfaction, which is associated with burnout, turnover intention, and intentions to leave the practice of medicine [20–22]. In addition, research has shown that physician satisfaction is associated with patients’ satisfaction [23, 24] as well as patients’ compliance with medical recommendations [25].

The lack of comparisons in delivering of buprenorphine versus extended-release naltrexone is notable given that implementation of the two medications may have some differences. For example, research has demonstrated major differences in the percentage of individuals who are successfully inducted onto the medication, with lower rates of successful induction for extended-release naltrexone than buprenorphine [26]. There is also some evidence of lower retention for extended-release naltrexone [15, 27], and that buprenorphine, but not extended-release naltrexone, being associated with lower odds of opioid-related overdose [28]. It is unknown if these challenges for initiation and retention translate into lower prescriber satisfaction with delivering extended-release naltrexone relative to buprenorphine satisfaction.

The aim of the current study was to expand on our prior work regarding buprenorphine satisfaction [19] by exploring the satisfaction of prescribers delivering buprenorphine as well as XR-naltrexone. In addition to considering satisfaction with delivering both types of MOUD, we sought to examine a new set of variables as potential correlates of MOUD satisfaction, including current MOUD patient census and prescribers’ perceptions of the larger organization. Delivery of MOUD requires physicians to develop additional skills and competence, as OUD can be a challenging condition to treat. Patient census was conceived as a proxy measure for competence and perceived effectiveness, as presumably physicians would only be willing to take on more patients if they felt they had the capacity and confidence to effectively treat them. Similarly, tenure in the addiction field and addiction-specific board certifications may serve as indicators of competence, as greater years of experience and gaining specialized training in addiction should be positively correlated with more developed clinical skills. Previous research has shown that clinicians’ perceived effectiveness is positively associated with job satisfaction [29]. Regarding the organizational environment, prior research on physician satisfaction has shown the importance of positive relationships among clinicians within organizations [30, 31] as well as the negative impacts of high job demands and burdens [29].

## Methods

### Sample and data collection

Data were drawn from a cluster randomized clinical trial (RCT) that sought to increase access to MOUD (NCT02926482) in 37 organizations in Florida, Ohio, and Wisconsin through a comparison of two sets of implementation strategies [32]. Within these 37 organizations, the study sample consisted of 61 treatment units within 31 specialty addiction treatment organizations and 14 treatment units within 6 health systems. Using procedures similar to a survey fielded in the first year of the study [19], during the third year of this project, the contact person for each organization participating in the RCT forwarded invitations to MOUD prescribers within these organizations to participate in a web-based survey. Multiple reminders were sent to potential participants about completing the survey, but no financial incentive for survey completion was provided. Forty-one respondents completed the survey from June 2019 to February 2020, representing 40 physicians and one nurse practitioner. The University of Wisconsin’s institutional review board approved all study procedures.

### Measures

Items measuring prescriber satisfaction with providing buprenorphine and XR-naltrexone treatment were adapted from the Physician Worklife Survey [33]. Among those providing each MOUD, five items asked about global satisfaction with their current buprenorphine work, and five items inquired about their current XR-naltrexone work. For each item, response options ranged from 1 (“strongly disagree”) to 5 (“strongly agree”). In addition to considering each item separately, mean scales of buprenorphine job satisfaction (α = 0.87) and XR-naltrexone job satisfaction (α = 0.89) were calculated, after reverse-coding the negative valence items.

The survey measured several prescriber characteristics as well as their perceptions of the organizational environment. Prescribers indicated whether they were waivered to prescribe buprenorphine (1 = yes, 0 = no) and whether their site provided XR-naltrexone (1 = yes, 0 = no). Waivered prescribers were asked about their current buprenorphine census (i.e., number of patients currently being treated with buprenorphine) and current waiver limit (i.e., 30 patients, 100 patients, or 275 patients). Using these two pieces of information, an additional variable was constructed regarding the percentage of the waiver currently used. For prescribers of XR-naltrexone, current census of patients being treated with XR-naltrexone was also reported. Prescribers were asked about the following addiction-focused board certifications: Addiction Medicine by the American Board of Addiction Medicine, Addiction Medicine by the American Board of Preventive Medicine, and Addiction Psychiatry by the American Board of Psychiatry and Neurology. Responses were coded into those with addiction board certification ( = 1) and those without certification ( = 0). Four items measured perceptions of the organizational environment, with responses ranging from 1 (“strongly disagree”) to 5 (“strongly agree”). These items focused on organizational commitment, positive relationships with clinical staff, work overload, and perceived respect by the organization. Demographic characteristics included age, gender, and race (0 = white, 1 = all others).

### Statistical analysis

First, descriptive statistics were calculated for all measures. Due to the small sample, analyses relied upon Pearson’s product-moment correlations between the measures of prescriber satisfaction and patient census, physician experience, and perceptions of the organizational environment. For the sub-sample who reported being waivered and prescribing XR-naltrexone, paired *t*-tests were used to test whether prescriber satisfaction differed between the two medications. Given the exploratory nature of the study, we did not correct for multiple comparisons. Data were collected during an organizational RCT in three states, so study condition of the prescribers’ organization was examined using independent samples *t*-tests and the three states were compared by one-way analysis of variance. There were no significant differences in buprenorphine satisfaction or XR-naltrexone satisfaction by study condition or by state (results not shown).

## Results

Among the 41 respondents, the average age was 51.0 (SD = 11.0), 37.5% (n = 15) were female, and the majority identified as white (70.7%, n = 29). About 37.5% (n = 15) were board certified in an addiction specialty. Nearly all of the respondents held the waiver to prescribe buprenorphine (92.7%, n = 38). Of the waivered prescribers, the most common waiver type was the 275-patient waiver (44.7%, n = 17); 31.6% held the 100-patient waiver (n = 12), and 23.7% held the 30-patient waiver (n = 9). The average census of current buprenorphine patients among waivered prescribers was 56.4 (SD = 57.0), but the average proportion of their waiver being used was just 37.4% (SD = 24.8). About 78.1% of the sample (n = 32) indicated that they provide XR-naltrexone for OUD, and for these prescribers, the average census of patients currently receiving XR-naltrexone for OUD was 20.7 (SD = 26.3).

Table 1 presents satisfaction with buprenorphine work among waivered prescribers and satisfaction with XR-naltrexone work among those offering this medication to treat OUD. As seen in Table 1, physicians were generally satisfied with their work in delivering these two types of MOUD. Satisfaction measures with positive valences (e.g., personally rewarding) had means near 4.0, indicating agreement, while items with negative valences (e.g., frustration) had means near 2.0, indicating disagreement. For the sub-sample of prescribers offering both medications (n = 31), paired t-tests indicated there was only one significant difference in the five items, such that prescribers more strongly endorsed buprenorphine work being a source of frustration (mean = 2.3, SD = 0.9) than XR-naltrexone work (mean = 2.0, SD = 0.8, *t* = -2.52, *p* = .02) (other results not shown).

**Table 1:** Descriptive statistics of physician satisfaction with buprenorphine (n = 38) and XR-naltrexone work (n = 32)

	Buprenorphine Work		XR-Naltrexone Work	
	Mean (SD)	% Agree or Strongly Agree (n)	Mean SD	% Agree or Strongly Agree (n)
I find my present work personally rewarding.	4.2 (0.7)	84.2% (32)	4.1 (0.9)	81.3% (26)
Overall, I am pleased with my work.	4.2 (0.6)	89.5% (34)	4.1 (0.8)	87.5% (28)
Overall, I am satisfied with my current practice.	4.1 (0.7)	83.8 % (31)	3.9 (0.8)	84.4% (27)
My current work is a major source of frustration.	2.2 (0.9)	13.2 % (5)	2.0 (0.8)	3.1% (1)
My work in this practice has not met my expectations.	2.1 (0.8)	7.9% (3)	2.1 (1.0)	12.5% (4)
Mean satisfaction scale	4.0 (0.6)		4.0 (0.7)	

*Notes.* Items were adapted from the Physician Worklife Survey (Williams et al., 1999), with response options ranging from 1 = strongly disagree to 5 = strongly agree. Negative valence items were reverse-coded for the mean scales

Bivariate correlations between satisfaction with buprenorphine work, prescriber characteristics, and perceptions of the organizational environment are presented in Table 2. Current number of buprenorphine patients was associated with all five satisfaction measures, such that prescribers with greater numbers of patients more strongly endorsed the positive valence questions and reported lower frustration and unmet expectations. However, the measure of the proportion of waiver used was not associated with any of the buprenorphine satisfaction measures. Regarding perceptions of the organizational environment, prescribers who would recommend the treatment center to colleagues more strongly endorsed the positive valence items while reporting lower frustration and unmet expectations. Good relationships with clinical staff were also associated with the satisfaction items, with the exception of the item regarding unmet expectations. Feeling overburdened by clinical responsibilities was only associated with one of the five satisfaction items (i.e., satisfied with current practice), while feeling more valued by the organization was correlated with greater endorsement of buprenorphine work being rewarding and lower endorsement of frustration. There were no differences in satisfaction with buprenorphine work by tenure in the addiction field or by addiction-specific board certification.

**Table 2:** Pearson’s product-moment correlations between patient volume, prescriber characteristics, organizational environment, and buprenorphine satisfaction

	I find my present buprenorphine clinical work personally rewarding.	Overall, I am pleased with my buprenorphine work.	Overall I am satisfied with my current buprenorphine practice.	My current buprenorphine work situation is a major source of frustration.	My work in this buprenorphine practice has not met my expectations.
Current number of buprenorphine patients	0.36*	0.39*	0.35*	-0.41*	-0.34*
Proportion of waiver used (i.e., current number divided by waiver limit)	0.03	0.12	0.30	-0.21	-0.28
Tenure in addiction treatment field	-0.16	-0.20	-0.14	0.14	0.04
Certified in an addiction specialty	0.09	0.20	-0.04	-0.11	0.07
I would recommend this treatment center to colleagues.	0.53***	0.59***	0.47**	-0.43**	-0.34*
I have a good relationship with the clinical staff of this organization.	0.62***	0.63***	0.41*	-0.45**	-0.28
I feel overburdened with my current clinical responsibilities at this organization.	-0.24	-0.25	-0.38*	0.31	0.27
I feel valued and respected by this organization.	0.36*	0.27	0.13	-0.35*	-0.23

*p < .05, **p < .01, ***p < .001 (two-tailed tests)

Next, the buprenorphine work satisfaction items were combined into a mean scale of overall satisfaction, and the prescriber characteristics and perceptions of the organizational environment were re-examined. Of the eight variables, four were significantly associated with overall buprenorphine work satisfaction. Prescribers reporting a greater number of patients (*r* = .46, *p* = .006), those who would recommend the center to others (*r* = .56, *p* < .001), and those reporting positive relationships with staff (*r* = .56, p < .001) reported significantly greater overall buprenorphine work satisfaction. Prescribers who more strongly endorsed feeling overburdened reported lower overall buprenorphine satisfaction (*r* = -.37, *p* = .02).

Table 3 presents bivariate correlations between the satisfaction items for XR-naltrexone work, patient volume, prescriber characteristics, and perceptions of the organizational environment. Overall, there were few significant correlations. The current number of XR-naltrexone patients was only correlated with the negative valence items, such that prescribers with more patients reported significantly lower levels of frustration and unmet expectations. Tenure in the addiction field was negatively associated with being pleased with and being satisfied with XR-naltrexone work, such that prescribers with greater tenure reported lower satisfaction. None of the organizational environment items were correlated with the items for XR-naltrexone work satisfaction. When the five items were combined into an overall scale of XR-naltrexone work satisfaction, none of the variables were significantly associated with this scale.

**Table 3:** Pearson’s product-moment correlations between patient volume, prescriber characteristics, organizational environment, and injectable naltrexone satisfaction

	I find my present injectable naltrexone clinical work personally rewarding.	Overall, I am pleased with my injectable naltrexone work.	Overall I am satisfied with my current injectable naltrexone practice.	My current injectable naltrexone work is a major source of frustration.	My work in this injectable naltrexone practice has not met my expectations.
Current number of injectable naltrexone patients	0.26	0.17	0.17	-0.40*	-0.37*
Tenure in addiction treatment field	-0.27	-0.45*	-0.38*	-0.03	0.31
Certified in an addiction specialty	0.21	0.19	0.01	-0.29	0.05
I would recommend this treatment center to colleagues.	0.23	0.14	-0.06	-0.29	-0.10
I have a good relationship with the clinical staff of this organization.	0.24	0.15	-0.09	-0.32	-0.13
I feel overburdened with my current clinical responsibilities at this organization.	0.11	0.01	0.08	0.02	-0.04
I feel valued and respected by this organization.	-0.08	-0.08	-0.12	-0.15	-0.07

*p < .05, **p < .01, ***p < .001 (two-tailed tests)

## Discussion

This study examined prescribers’ satisfaction with providing two types of MOUD, buprenorphine and XR-naltrexone. We found that prescribers are generally satisfied with providing these two MOUDs, which is important since prescriber dissatisfaction can lead to turnover and impact intentions to leave the profession [34, 35]. Ratings for the two MOUDs were remarkably similar, which was somewhat surprising given the induction challenges [26] and shorter average retention that occurs with extended-release naltrexone [15, 27]. However, the correlates of satisfaction differed between the two medications, in that patient volume and some of the organizational context measures were associated with buprenorphine work satisfaction, but none of the variables were associated with XR-naltrexone work satisfaction.

One notable finding was that the number of patients was positively correlated with prescriber satisfaction with delivering buprenorphine treatment. This magnitude of the association between census and the scale of buprenorphine satisfaction represented an effect size in the range of medium to large [36]. Questions remain about the direction of causality. It may be that treating more patients is an indicator of proficiency in or penchant for delivering this type of MOUD, which may positively impact satisfaction in treating this population. In other types of medical care, such as surgical procedures, a positive relationship between patient volume and patient outcomes is well-established [37, 38]. Treating more patients also provides more opportunities to observe patients entering remission and experiencing positive changes in their lives, which may also be a source of satisfaction. It may also be that prescribers who are more satisfied are more willing to take on additional patients. Future research should aim to replicate this finding in a larger sample with longitudinal data to help to better understand this association.

For buprenorphine work satisfaction, the associations regarding positive relationships with clinical staff and overly burdensome work demands align with prior research on physician satisfaction [29–31]. These correlations represented medium to large effect sizes [36].What is puzzling, however, was that these measures of the organizational context were not associated with XR-naltrexone work satisfaction. One potential explanation may be sample size, in that fewer prescribers offered XR-naltrexone than buprenorphine. However, it may also be that there are differences in how these two types of MOUD are implemented in clinical settings, which may then have implications for if or how the organizational context may matter in terms of prescriber satisfaction. Future research using qualitative semi-structured in-depth interviews with prescribers may be an important method for better understanding how organizational context intersects with satisfaction in delivering different types of MOUD. In the current study, qualitative data collection focused on the issue of MOUD capacity-building from the perspective of organizational champions of MOUD who were most often in administrative positions [39]. The qualitative interviews revealed novel facilitators and barriers that differentiated organizations that made continuous improvements in MOUD capacity from those that did not. This ability to identify innovative factors inductively is a key strength of qualitative methodology and points to how in-depth interviews with prescribers could similarly lead to novel insights regarding satisfaction with delivering MOUD.

The study also provides insights into how the provision of MOUD might impact prescriber retention patterns. For prescribers, higher volumes of buprenorphine patients correlated with higher satisfaction levels. Simply encouraging prescribers to prescribe more buprenorphine will not likely lead to higher satisfaction by itself. Future research should seek to explore what leads to an individual physician wanting to prescribe buprenorphine to more patients and to create those working conditions that could lead to greater use of buprenorphine as well as greater satisfaction. For example, if a prescriber is expressing concerns regarding their confidence in using buprenorphine, there may be value in engaging them with an Extension for Community Healthcare Outcomes (ECHO) session or a peer mentor. The positive correlation between strong relationships with other clinical team members and buprenorphine satisfaction may indicate that a multi-discipline care model helps to make delivering buprenorphine treatment more meaningful and less burdensome. Our findings about XR-naltrexone satisfaction and tenure in the field suggest that perhaps use of XR-naltrexone could be encouraged for prescribers who are earlier in their career. For those more advanced in their career, it may be that additional supports are needed to assist them with using this MOUD and appreciating its benefits, as those with greater tenure in the addiction field reported lower XR-naltrexone satisfaction.

Although this sample of prescribers is small, the findings aligned with a number of studies that have reported that most prescribers of buprenorphine are not fully utilizing their capacity based on their waiver type [14, 40–44]. A recent analysis documented that a small subset of buprenorphine prescribers (~5 %) were responsible for nearly half of US prescriptions over a two year period, and even these high volume prescribers averaged about 124 patients per month, which was well below the 275 patient cap [45]. In this sample, prescribers were on average using only about one-third of their capacity. Prescribers treated even fewer patients with injectable naltrexone, a finding that is consistent with other studies that have examined insurance claims data on utilization [15]. Unused MOUD treatment capacity is concerning given the scope of the ongoing opioid epidemic. Additional research is needed to further elucidate the factors associated with low utilization.

Several limitations to this study should be noted. First, the sample was quite small, and the prescribers were recruited from only three states. Our data collection strategy relied upon a key point of contact forwarding the survey invitations to prescribers, making it difficult to ascertain a response rate. A separate survey of organizational characteristics indicated a total of 65 waivered prescribers worked in these organizations, and we received responses from 38 waivered prescribers (58%). It is unknown if dissatisfied prescribers were less likely to respond to the survey. Also, the findings are from a cross-sectional survey which cannot establish causality. In addition, this study was conducted in the context of an RCT focused on expanding MOUD, so the organizations recruited as sites may have cultures that are more supportive of MOUD which may have implications for physician satisfaction. Finally, only a limited number of constructs were included in the survey. Additional domains may be associated with prescriber satisfaction, such as challenges in obtaining reimbursement from insurers and stress experiences from concerning patient behaviors (e.g., misuse and diversion of buprenorphine, treatment dropout). Personality factors may also be correlated with prescriber satisfaction. Future research should also consider whether some of the factors that are associated with general job satisfaction are also correlated with prescribers’ satisfaction in providing MOUD.

While future research should examine these issues in a larger, more generalizable sample, the study points to potentially important directions for future research. Continuing to examine additional factors associated with prescriber satisfaction with MOUD may identify directions for intervention development, including strategies to increase MOUD use and foster prescriber retention. Furthermore, qualitative data collection may help to elucidate why the factors associated with satisfaction seemed to differ between the two types of medication.

## Conclusions

In the context of the opioid epidemic, there are ongoing concerns of shortages of prescribers of MOUD [46–48], so satisfaction among those providing these evidence-based practices is important. This sample of prescribers reported general satisfaction with delivering buprenorphine and XR-naltrexone, and their ratings did not differ between these two types of MOUD. There may be value in encouraging prescribers with small caseloads to consider expanding their MOUD practice, as our findings suggest there may be a benefit for prescriber satisfaction from treating more patients with MOUD, although more research is needed to confirm this finding. Managers of clinics should attend to supporting a positive organizational climate, as measures of organization context were associated with satisfaction in delivering buprenorphine. Future research is needed to examine multivariate models of satisfaction, to determine whether MOUD dissatisfaction is associated with prescribers’ de-implementing this service, and to develop interventions that may reduce dissatisfaction.

## Data Availability

Given the small sample size, the dataset for the current study is not publicly available in an effort to protect participant confidentiality. However, they are available from the corresponding author on reasonable request.

## Abbreviations

COVID-19: coronavirus disease
ECHO: Extension for Community Healthcare Outcomes
MOUD: medication for opioid use disorder
OUD: opioid use disorder
RCT: randomized clinical trial
XR: extended-release
